# A simple completion of Fisher’s fundamental theorem of natural selection

**DOI:** 10.1002/ece3.6918

**Published:** 2020-12-27

**Authors:** Alan Grafen

**Affiliations:** ^1^ Zoology Department Oxford University Oxford UK

**Keywords:** additive genetic component, fitness definitions, formal Darwinism, fundamental theorem of natural selection, partial change in mean fitness, quantitative genetics

## Abstract

Fisher's fundamental theorem of natural selection shows that the part of the rate of change of mean fitness that is due to natural selection equals the additive genetic variance in fitness. Fisher embedded this result in a model of total fitness, adding terms for deterioration of the environment and density dependence. Here, a quantitative genetic version of this neglected model is derived that relaxes its assumptions that the additive genetic variance in fitness and the rate of deterioration of the environment do not change over time, allows population size to vary, and includes an input of mutational variance. The resulting formula for total rate of change in mean fitness contains two terms more than Fisher's original, representing the effects of stabilizing selection, on the one hand, and of mutational variance, on the other, making clear for the first time that the fundamental theorem deals only with natural selection that is directional (as opposed to stabilizing) on the underlying traits. In this model, the total (rather than just the additive) genetic variance increases mean fitness. The unstructured population allows an explanation of Fisher's concept of fitness as simply birth rate minus mortality rate, and building up to the definition in structured populations.

## Graphical abstract

Fisher's model of the total change in mean fitness incorporates density dependence and a deteriorating environment along with the effect of natural selection provided by the fundamental theorem. Here, a quantitative genetic version of Fisher's model is provided, which illuminates the fundamental theorem. In particular, it has not been recognized before that the theorem treats stabilizing selection and directional selection on the underlying traits differently.

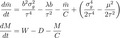

## 1 INTRODUCTION

The absence of the fundamental theorem from the unfolding of biological ideas is starkly illustrated by its omission from Huxley (1942)'s defining *Evolution: The Modern Synthesis*. Many of Fisher's result are cited, but the result Fisher believed stood comparison with the Second Law of Thermodynamics (Fisher, 1930, page 30) is not mentioned at all in the founding work of modern biology's fusion of Darwinism and Mendelism. Here, we help to incorporate it into today's thinking by showing how the theorem encourages a dissection of the forces affecting mean fitness, bringing in density dependence and deterioration of the environment, and explaining Fisher's concept of fitness.

After passing through various other stages, the consensus among mathematical population geneticists is currently that the theorem is true mathematically, but that any biological significance has yet to be uncovered, following the line of Price (1972b). The main reason given is that the left‐hand side of the theorem is a *partial* change in mean fitness and not the total change. Price's point has been generally adopted, including by Ewens (1989, 1992, 2004) and Edwards (2002, 2014). These authors see a real point in a formula for the *total* mean fitness, but Fisher's theorem is inadequate by that standard, and are unconvinced that Fisher's partial change is meaningful. Grafen (2018) articulates the case (expressed characteristically tersely by Fisher himself) that the partial change is a natural and powerful way to capture the component of change in mean fitness that is due to natural selection, though Lessard and Ewens (2019) remain unconvinced. Fisher's significant motivation was to provide a mathematical version of Darwin's argument that natural selection is an improving process, which evidently requires a way to isolate the component due to natural selection: those who oppose Fisher's suggestion offer none of their own (e.g., Charlesworth, 1994; Walsh & Lynch, 2018).

On the other hand, empirical biologists and less mathematically technical modelers continue to draw inspiration from the general gist of the theorem. There is currently a new surge in attempts to measure fitness in natural populations, and also to measure the additive genetic variance in fitness at least partly because of its role as the right‐hand side of the fundamental theorem (Bonnet et al., 2019; Burt, 1995; Hendry et al., 2018; Hunter et al., 2019; Reid et al., 2019). The theorem continues to be explained and discussed in abstract terms (Frank, 1997, 1998, 2012; Queller, 2017, 2020). The current paper contributes a precise mathematical model to add to this more positive literature on the fundamental theorem. The kinds of reasons for this divergence between different biologists on the fundamental theorem are considered by Welch (2017).

As the theorem provides a *partial* change, one natural approach aimed to "complete" the fundamental theorem by finding an expression for the total change in fitness. This has most effectively been undertaken, so far as complex genetic effects such as linkage and epistasis are concerned, by Nagylaki (1993). Earlier, Fisher had himself provided such a "completion" in 1930, ten pages after the fundamental theorem, incorporating effects of the *eco‐evolutionary* forces of deterioration of the environment and density dependence. His primary motivation seems to have been to defend the fundamental theorem from the charge that it predicts that mean fitness always increases, which would be contrary to fact and to logic. More specifically, he proposed a model in which the rate of change in mean fitness *M* equals the sum of three terms. The additive genetic variance in fitness *W* is the partial effect calculated by the fundamental theorem. Fisher subtracts a term *D* for the deterioration of the environment, and a further term *M*/*C*, where *C* is a constant, for density‐dependent effects. This third term is initially puzzling, as one would expect the population size rather than *M* itself to appear in such a term, but –*M*/*C* turns out to represent the Gompertz form of density dependence (Kirkwood, 2015). Thus, Fisher (1930, page 42) arrives at

(1) $$\frac{\mathrm{dM}}{\mathrm{dt}}=W‐D‐\frac{M}{C}.$$

This formula has received rather little attention in the literature, but it clearly shows Fisher's view that the fundamental theorem provides only part of the change in mean fitness and that other forces will act simultaneously. I will refer to this model as the "MWCD model." The fundamental theorem itself operates at a very high level of abstraction, making few assumptions (Ewens, 1989, 2004; Grafen, 2015a; Price, 1972b): Any "completion" will inevitably involve a much more specific model with restrictive assumptions.

Fisher makes the unrealistic assumptions that both *W* and *D* do not change over time in his analysis of this equation (though of course not in his derivation of the fundamental theorem itself). In Section 2, a quantitative genetic version of the model is developed that relaxes these assumptions. This extension allows us to make in Section 3 some new points about the operation of natural selection, and thus demonstrate the utility of separating out the total change into different components. For example, the model will show that Fisher's identification of the additive genetic variance with the effect of natural selection is not quite right.

This unearthing of Fisher's ideas and models thus has contemporary relevance in still allowing new points to be made. The discussion in Section 4 explains Fisher's concept of fitness, which turns out to be very simple and closely connected to contemporary ideas for him and for us.

The view proposed here is that the fundamental theorem is essentially a suggestion of two definitions, with the statement of the theorem a powerful result available if we adopt them both. The first definition is of fitness, and the second is of "due to natural selection," which explains how to isolate one component of the total change (for a full exposition, see Grafen, 2015a, 2015b, 2018). Under these definitions, the theorem tells us that the part of the change in mean fitness that is due to natural selection equals the additive genetic variance in fitness, which implies that natural selection is an improving process *and* tells which quantity is improved by it. Grafen (*loc. cit*.) argues that this link is invaluable in understanding biological design and formalizing Darwin's core argument.

## 2 THE MODEL DERIVED

This section's goal is to construct the simplest quantitative genetic (QG) model that allows us to track mean fitness over time, as well as the variance of fitness divided into various components, and that incorporates a changing environment and density dependence. Our starting point is a model of Lynch et al. (1991), extended to allow population size to change over time. We track a population of variable size *n* with a quantitative trait *x* = *x_g_* + *x_e_* that affects fitness. Assume that the environmental component *x_e_* has a normal distribution with zero mean and variance $\sigma_{e}^{2}$ conditional on the genetic component *x_g_*, independently within each individual. The genetic component is distributed across the population as *n* times a normal distribution:

(2) $$f\left(x_{g}\right)=\frac{n}{\sigma_{g}\sqrt{2}}\exp‐\left(\frac{1}{2}{\left(\frac{x_{g}‐\bar{x}}{\sigma_{g}}\right)}^{2}\right).$$

The relative growth rate of the subpopulation at *x*, written as *m*(*x*) and termed "fitness," is modeled by

(3) $$m\left(x\right)=r‐\frac{{\left(x‐\hat{x}\right)}^{2}}{2\tau^{2}}‐\frac{\log\;n‐\log\;n_{0}}{C},$$

with an intrinsic growth rate *r*, a quadratic term that implements natural selection by penalizing the deviation of *x* from the optimum $\hat{x}$ with the inverse strength of selection represented by τ, and minus a Gompertz density‐dependent term $\frac{\log n‐\log n_{0}}{C}$(for a review, see Kirkwood, 2015) that diminishes all growth rates equally as n increases. The change in distribution of $x_{g}$ depends on the mean fitness of individuals with a given value of $x_{g}$, averaging over the distribution of $x_{e}$, say $m_{g}\left(x_{g}\right)$, and Equations (11) and (12) in the Appendix show that

$$\frac{1}{f}\frac{df}{dt}=m_{g}\left(x_{g}\right)=r‐\frac{\sigma_{e}^{2}}{2\tau^{2}}‐\frac{{\left(x_{g}‐\hat{x}\right)}^{2}}{2\tau^{2}}‐\frac{\log\;n‐\log\;n_{0}}{C}.$$

Then, the rate of change of mean and variance of $x_{g}$, allowing a supply $\mu^{2}dt$ of new mutational variance, is as follows,

$$\frac{d\bar{x}\left(t\right)}{dt}=\left(\hat{x}‐\bar{x}\right)\frac{\sigma_{g}^{2}}{\tau^{2}}\;\frac{d\sigma_{g}^{2}\left(t\right)}{dt}=\mu^{2}‐\frac{\sigma_{g}^{4}}{\tau^{2}}.$$

These equations were first found by (Lynch et al. (1991, Equations (7) and (8) on page 1,304) in a very similar model, and are not reproved here. Thus, the change in the variance of $x_{g}$ is negatively related to its magnitude, and it approaches zero only as the variance itself approaches its equilibrium value. Therefore, $\sigma_{g}^{2}$ asymptotes to its equilibrium value. The independence of the second‐order process is a standard observation in this area (Lande, 1976).

Again following one of Lynch et al.'s directions, assume that the optimal value changes linearly with time, to emulate continuing environmental change, formally ${\hat{x}}_{t}={\hat{x}}_{0}+\lambda t$. For ease of description, we assume λ>0, though clearly the sign could be switched with no substantive consequence. Then, defining $b=\hat{x}‐\bar{x}$ to stand for how far the population lags behind the optimum, the system of ordinary differential equations in $\left(b,\sigma_{g}^{2}\right)$, is

(4) $$\frac{d\sigma_{g}^{2}\left(t\right)}{dt}=\mu^{2}‐\frac{\sigma_{g}^{4}}{\tau^{2}}$$

(5) $$\frac{db}{dt}=\lambda ‐b\frac{\sigma_{g}^{2}}{\tau^{2}}.$$

The current treatment adds to Lynch et al.'s that population size n varies, and the equation for fitness implies, as shown in the Appendix's Equations (12) and (11), that

$$\frac{d\;\log\;n}{\mathrm{dt}}={\text{ave}}_{f}m=r‐\frac{\sigma_{e}^{2}+\sigma_{g}^{2}+b^{2}}{2\tau^{2}}‐\frac{\log\;n‐\log\;n_{0}}{C},$$

Note the use of Price (1972a)'s notation for statistical operators over the population (see also Grafen, 2015b), so that ave*_f_ m_g_* is the *f*‐weighted average of $m_{g}$, and we will later use var*_f_ m_g_* as the corresponding variance. This completes the set of three differential equations in the variables $\left(\sigma_{g}^{2},b,n\right)$. The motion of b does not depend on n, so the system moves to an equilibrium with values

(6) $${\tilde{\sigma }}_{g}^{2}=\mu \tau$$

(7) $$\tilde{b}=\lambda \frac{\tau }{\mu }$$

(8) $$\tilde{n}=n_{0}\;\exp\left\{C\left(r‐\left(\frac{\sigma_{e}^{2}}{2\tau^{2}}+\frac{\mu }{2\tau }+\frac{\lambda^{2}}{2\mu^{2}}\right)\right)\right\}.$$

This completes the development of the model, and the interpretation of new aspects takes place in the next section. Note that the equilibrium variance is larger if there is more mutation, and larger if the strength of selection is weaker, both of which make intuitive sense. The chronic gap increases as the rate of environmental deterioration is larger, and as selection is weaker, and it decreases as the mutational input increases. All of these patterns are to be expected, and conform to the results of Lynch et al. (1991).

## 3 INTERPRETATION OF THE MODEL

This section compares the new model's formula for the total rate of change of mean fitness with Fisher's original, and interprets both. I have been unable to find any substantive discussion of Fisher's model in the literature, probably because the fundamental theorem on which it is based was so misunderstood, and because the Gompertz form of density dependence creates an initial difficulty in understanding the equation. The original and new forms are

(9) $$\frac{d\bar{m}}{dt}=\frac{b^{2}\sigma_{g}^{2}}{\tau^{4}}‐\frac{\lambda b}{\tau^{2}}‐\frac{\bar{m}}{C}+\left(\frac{\sigma_{g}^{4}}{2\tau^{4}}‐\frac{\mu^{2}}{2\tau^{2}}\right)$$

$$\frac{dM}{dt}=W‐D‐\frac{M}{C}.$$

The left‐hand sides are equal, as Fisher's M and our $\bar{m}$ are both mean fitness of the population. On the right‐hand side, the first terms correspond directly, as Equation (14) shows $\frac{b^{2}\sigma_{g}^{2}}{\tau^{4}}$ to be the additive genetic variance in fitness. Fisher's value was assumed fixed, but the QG model allows the underlying genetic trait to evolve in the standard way, and so it changes over time as the gap between mean and optimum (b) changes, *and* as the genetic variance $\sigma_{g}^{2}$ in the underlying trait varies. This first equivalence therefore shows that Fisher's assumption of constancy did no harm.

The second terms on the right‐hand side also correspond directly, as Fisher's *D* is the deterioration in mean fitness due to changes in the environment, and $\frac{\lambda b}{\tau^{2}}$ is the only term containing λ. Again, Fisher assumed D was fixed, but the QG model allows it to vary, and shows it is proportional to the gap b. This implies that if the gap is negative, meaning that environmental change is moving the population mean *toward* the optimum, then the deterioration is negative and environmental change is having a positive effect on mean fitness, just as one would expect. (The additive genetic variance does not become negative in this case, because it is proportional to the square of the gap.) Again, apart from identifying a circumstance in which the sign can switch, the analysis shows that Fisher's formula was correct despite his assumption of constant D. The third terms also correspond directly and reflect the Gompertz density dependence, which tends to increase a negative mean fitness and decrease a positive mean fitness.

An exciting difference between the formulae appears in the final term of Equation (9), which is missing in Fisher's version. The positive term in the difference is the nonadditive genetic variance in fitness, which depends on the variance in the underlying genetic trait. From this is subtracted the reduction in mean fitness that arises through the continuing mutational input—this effect arises because spreading the genetic trait will move some of the distribution toward the optimum, and some away, but on balance, it results in a reduction in mean fitness. The interpretation is simply that mutational input reduces fitness by spreading the distribution, and nonadditive genetic variance increases fitness by tightening it up.

How does this point affect the fundamental theorem itself? If we accept Fisher's definition of "due to natural selection" for the left‐hand side, then no change is necessary, as the derivation is correct. That definition includes only change due to changing gene frequencies, which correspond to directional selection. Stabilizing selection is a matter of linkage disequilibrium, with a tendency to place strong positive alleles in the same individuals as strong negative alleles. However, selection *does* bring about linkage disequilibrium, in a way that in turn *does* increase fitness. The simplest reaction is to understand that the left‐hand side of the fundamental theorem is "the rate of change in mean fitness due to the directional effects on traits brought about by natural selection," and recognize that there is a systematic force due to selection that is not included. It would be impossible to include that force at the level of generality of the fundamental theorem, as the minimal model that produces the theorem lacks necessary genetic details. It is also important to recognize that any "completion" of the fundamental theorem, aiming at the total change in mean fitness, such as the current model, must include these effects of stabilizing selection and nondirectional mutation. Mutations that are directional on underlying traits, which are typically assumed outside quantitative genetic models, are already included in the theorem. A question of potential interest to philosophers of biology is to ask whether Darwin's arguments should be thought of as applying only to directional natural selection, excluding its purifying component, or whether this restriction applies only to Fisher's formalizing of it.

Thus, the new model helps us understand Fisher's model better. It is curious that the QG model shows that the total genetic variance in fitness can be regarded as increasing mean fitness, and this may link to a similar finding by Morrissey and Bonnet (2019). The important methodological conclusion is that pursuing the idea of analyzing the change of mean fitness into components, corresponding to different causes, continues to improve our understanding of natural selection.

One further point is worth making about the QG model. The role of mutation is nicely displayed in the formula for the equilibrium population size in Equation (8). Mutation helps the population mean keep up with the optimum, and so increases population size, as reflected in the term $\frac{\lambda^{2}}{2\mu^{2}}$, which enters with a negative sign into the exponent. The larger the ratio of rate of change to mutational input, the smaller the population size. Mutation also tends to spread out the population, increasing the variance of the trait, and this effect reduces the mean fitness, as reflected in the $\frac{\mu }{2\tau }$ term, also with a negative sign: The higher the ratio of mutation rate to strength of the stabilizing selection, the lower the population size.

The QG model is clearly very special, as it assumes distributions are all normal and selection is Gaussian. I concur with Morrissey and Bonnet (2019) that further work could usefully explore how varying these special assumptions affects the conclusions. For example, how widely is it true that the whole genetic variance in fitness contributes to increasing the mean fitness? Does the balance between additive genetic variance and deterioration of the environment hold with other patterns of changing environment, and with genetic architectures that do not conform to QG? One further assumption within the QG model is that there is only one underlying trait. Allowing multiple traits, but having them combine additively to make a single "summary trait" that then determined fitness through the same quadratic machinery as the model here, would make little difference except that linkage disequilibrium between the traits would cause complications; allowing nonadditivity would clearly represent a significant increase in biological complexity, and there are reasons this is rarely attempted in quantitative genetics.

The analysis of the total change in mean fitness shows the significance of Fisher's model in contextualizing the fundamental theorem by adding environmental change and density dependence, but also refines our understanding of the fundamental theorem itself, and so improves our formal understanding of Darwin's arguments. The next section considers Fisher's definition of fitness in light of the MWCD model.

## 4 DISCUSSION

Fisher's concept of fitness has often seemed obscure. This crucial concept appears on both the left‐ and right‐hand sides of the fundamental theorem, but Fisher offers no explicit definition. Even when I presented a fully explicit derivation of the theorem in precisely Fisher's context (Grafen, 2015a), or in a generalization (Grafen, 2015b), the definition of fitness was complicated by the class structure of the model. The quantitative genetic version of the MWCD model from Section 2 has no class structure, and so offers an opportunity for a simple explanation of Fisher's idea. The concept is explained first, and then, the justification will be given for regarding this as Fisher's definition.

In order to match other uses, we start afresh in defining notation. Fisher's fitness is an individual‐level quantity, so we represent the fitness of individual i as $m_{i}$. In a nonstructured model, it is simply

$$m_{i}=b_{i}‐\mu_{i},$$

where $b_{i}$ is the instantaneous birth rate, and $\mu_{i}$ is the instantaneous death rate. This is a standard formulation in continuous time unstructured models, and Fisher may easily have come across very similar formulae in Equations (5) and (6) in Chapter IX of Lotka (1925): though Lotka does not interpose the term or concept of "fitness" between the rate of change of population size and the difference between birth and death. $m_{i}$ represents the net rate of growth of i's lineage, with the crucial qualification that sexual diploidy implies there are no separate lineages. A value of 0 is neutral, and an individual's genes are diminishing in frequency with negative values, and increasing with positive values. At a population level, the population growth rate is the mean value of the $m_{i}$. This neat connection between the average of an individual‐level quantity and the population growth rate was already used by Lotka. Fisher's special addition was to use this definition, by itself entirely straightforward in an asexual context, to obtain meaningful results under sexual diploidy and a general genetic architecture.

It is worth pointing out that the property that the average of an individual quantity equals the population growth rate emerges from the Gaussian fitness function first employed within quantitative genetics by Latter (1960), who himself attributes it to Haldane (1954), and subsequently much used (e.g. Lande, 1976; Lande et al., 2009, 2017; Lande & Shannon, 1996; Rousset, 2004; Rousset & Ronce, 2004), and is of central significance in evolutionary rescue models (Anciaux et al., 2018; Gomulkiewicz & Holt, 1995). The special genetic architecture assumed in quantitative genetics is the reason for this similarity with asexual models.

Let us now make the promised return to showing that we have in fact been discussing Fisher (1930)'s definition of fitness. We have to remove age from the second formula on his page 30, namely

$$dv_{x}‐\mu_{x}v_{x}dx+b_{x}v_{0}dx=mv_{x}dx,$$

where x is a subscript indicating age. $v_{x}$ is the per‐capita reproductive value, $b_{x}$ the birthrate, and $\mu_{x}$ the death rate, all appropriate to age x. An informal way to remove age from the model is to set reproductive value to 1 and drop the age subscript. A more formal way is to note that without age structure, b and μ cannot depend on x. It then follows from Fisher (1958)'s formula for reproductive value (preferred to the first edition because of the erroneous omission of $v_{0}$ there) that $v_{x}$ also does not depend on x, and is positive. We arrive at the same point as the less formal approach, which yields

(10) b‐μ=m,

where it is important to note that these are three population average quantities. Fisher then introduced breeding values (which he calls "genetic expectations") on pages 30–34, in relation to stature as an example of a quantitative trait. Later on page 34, he wrote

The definitions [sc. of average excess, average effect and genetic expectation] given above may be applied to any characteristic whatever; it is of special interest to apply them to the special characteristic m which measures the relative rate of increase or decrease. (p34, the brackets are added)

The reader's conundrum is that Fisher had not defined his m as an individual property, but only in terms of averages over age groups. Grafen (2015a) proposed that the obvious way for Equation (10) to deliver an individual‐based $m_{i}$ for individual i is to suppose that birth rate and death rate are individual characters $b_{i}$ and $\mu_{i}$, and that $m_{i}=b_{i}‐\mu_{i}$. The average property then holds if we define b, μ, and m as the averages of the respective individual variables. Fisher did not spell this step out, but it seems simple enough, and Grafen showed that it leads to a coherent mathematical development that recovers all of Fisher's results in that section, including the fundamental theorem. To adopt this suggestion that $m_{i}=b_{i}‐\mu_{i}$ is to regard the definition used earlier in this section as indeed that of Fisher.

Thus, Fisher's definition of fitness turns out to be very simple in unstructured populations. The first sophistication in age‐structured populations is that an individual has a fitness at each moment of time: while this is also true in the unstructured model, that instantaneous fitness remains constant over time, while with age structure, an individual's fitness changes over time. Thus, fitness is not a "tombstone evaluation" of an individual's reproductive success, but a dynamic quantity indicating success at a particular moment. Essentially, at each moment, its own possibly surviving self is treated as a special kind of offspring. The second sophistication is that the gain through reproduction and loss through mortality of individuals must be weighted by their *reproductive value*, so that $b_{x}v_{0}‐\mu_{x}v_{x}$ is still birth rate minus mortality rate, but takes into account the influence on the future gene pool, and is not just a head count (this quantity is sometimes called "Williams’ reproductive value" Grafen, 2015a; Grafen, 2020). The third and final sophistication is to divide this quantity by the reproductive value an individual expects by virtue of its age, yielding fitness for individual i of age x as

$$m_{i}=\frac{b_{i}v_{0}‐\mu_{i}v_{x}}{v_{x}}.$$

The division by $v_{x}$ has the consequence that the average fitness of each age class is the same, and equal to the Malthusian parameter. Thus, fitness is interpreted as how much an individual spreads her alleles, compared with others of the same age, with a value of zero meaning her alleles are not increasing in number at all. Computationally, we can regard $b_{x}v_{0}‐\mu_{x}v_{x}$ as counting the change in gene copies, and realize that we need to add this up over individuals to find the success of an allele; but given we are going to follow Fisher's advice to weight fitness by the individual's reproductive value when taking the average, we need to divide $b_{x}v_{0}‐\mu_{x}v_{x}$ by $v_{x}$ so that once weighted, we are indeed adding up the numerators. See Grafen (2015a, 2015b) for more discussion, or a much less technical account by Grafen (2020).

The rest of biology has continued to use the idea of birth rate minus death rate as rate of increase whenever the assumptions are plausible, for example, clonal populations and quantitative genetics. The benefit of using the fundamental theorem is obtaining the desirable quantification of Darwin's central argument that natural selection is an improving process, and discovering what quantity is improved. The cost of using it is (a) adopting Fisher's very reasonable definition of fitness, shown above to be simple and direct, and (b) adopting Fisher's understanding of which part of the change in the mean of a trait should be counted as "due to natural selection," argued above to be a device for obtaining a simple unifying concept under a general genetic architecture and sexual diploidy.

To sum up, the MWCD model has been expanded and shown largely to confirm Fisher's conclusions even when his assumptions of constant W and constant D are relaxed, but we now understand the fundamental theorem to deal not with all of natural selection, but to exclude stabilizing selection and deal only with natural selection that is *directional* on the underlying traits. The opportunity to encounter Fisher's fitness in an unstructured model has allowed its very simple definition as birth rate minus mortality rate to be explained. Fisher was the first to "complete" the fundamental theorem by adding extra terms to determine the *total* rate of change in mean fitness. We have seen that this exercise reinforces the purpose of the fundamental theorem, which is to isolate from the total change a component to be regarded as "due to natural selection." Without such a quantification, it is impossible to formalize Darwin's main point that natural selection is an improving process. Generalizations about natural selection need to be able to isolate this component, and say what quantity is improved: Fisher's theorem shows us how to do both, and the logic of his age‐structured model can be straightforwardly extended to general structured populations (Grafen, 2015b).

## CONFLICT OF INTEREST

I have no competing interests.

## Data Availability

This paper contains no data.

## Appendix

One basic result and the new results are derived here. The average growth rate of individuals with a given value of $x_{g}$ is the average over the environmental component of $m\left(x\right)$ given in Equation (3). Splitting the square ${\left(x‐\hat{x}\right)}^{2}$ into

$${\left(x_{g}+x_{e}‐\hat{x}\right)}^{2}={\left(x_{g}‐\hat{x}\right)}^{2}+2x_{e}\left(x_{g}‐\hat{x}\right)+x_{e}^{2},$$

and taking the average over the distribution of $x_{e}$, relying on the conditional independence of $x_{e}$ and its zero mean, gives the average value as

$${\left(x_{g}‐\hat{x}\right)}^{2}+\sigma_{e}^{2}$$

which we will use below in the expression for $m_{g}\left(x_{g}\right)$

The weighted averages will be with respect to the distribution of the trait's genetic component $x_{g}$, denoted f and defined in Equation (2). Note that f integrates to the population size n, and not to 1. The average fitness $\bar{m}$ (obtained by averaging over the average fitness $m_{g}$ for each $x_{g}$) is

(11) $$\begin{array}{cc} \bar{m} & =ave_{f}\;m_{g}=ave_{f}\left(r‐\frac{\sigma_{e}^{2}}{2\tau^{2}}‐\frac{{\left(x_{g}‐\hat{x}\right)}^{2}}{2\tau^{2}}‐\frac{\log\;n‐\log\;n_{0}}{C}\right)\\ & =r‐\frac{\log\;n‐\log\;n_{0}}{C}‐\frac{\sigma_{e}^{2}}{2\tau^{2}}‐\frac{\sigma_{g}^{2}+b^{2}}{2\tau^{2}} \end{array}$$

The per‐capita change in population size equals this value, as

(12) $$\begin{array}{ll} n & =\int f\mathrm{dx},\\ \frac{1}{n}\frac{\mathrm{dn}}{\mathrm{dt}} & =\frac{\int \frac{\mathrm{df}}{\mathrm{dt}}dx}{\int \mathrm{fdx}}=\frac{\int \frac{1}{f}\frac{\mathrm{df}}{\mathrm{dt}}dx}{\int \mathrm{fdx}}=\frac{\int m_{g}fdx}{\int \mathrm{fdx}}=ave_{f}\;m_{g}. \end{array}$$

An expression for the genetic variance of fitness *m_g_* is found by expanding as follows,

(13) $$\begin{array}{ll} {\text{var}}_{f}\;m_{g} & ={\text{var}}_{f}\left(r‐\frac{\sigma_{e}^{2}}{2\tau^{2}}‐\frac{(x_{g}‐\hat{x}){\;}^{2}}{2\tau^{2}}‐\frac{\log\;n‐\log\;n_{0}}{C}\right)\\ & ={\text{var}}_{f}\left(‐\frac{(x_{g}‐\bar{x}){\;}^{2}}{2\tau^{2}}‐2\frac{\left(x_{g}‐\bar{x}\right)\left(\bar{x}‐\hat{x}\right)}{2\tau^{2}}‐\frac{(\bar{x}‐\hat{x}){\;}^{2}}{2\tau^{2}}\right)\\ & =\frac{{\text{var}}_{f}\left((x_{g}‐\bar{x}){\;}^{2}+2\left(x_{g}‐\bar{x}\right)\left(\bar{x}‐\hat{x}\right)\right)}{4\tau^{4}}\\ & =\frac{{\text{var}}_{f}\left((x_{g}‐\bar{x}){\;}^{2}\right)+4b^{2}{\text{var}}_{f}\left(x_{g}‐\bar{x}\right)‐4b{\text{cov}}_{f}\left((x_{g}‐\bar{x}){\;}^{2},\left(x_{g}‐\bar{x}\right)\right)}{4\tau^{4}}\\ & =\frac{{\text{ave}}_{f}\left((x_{g}‐\bar{x}){\;}^{4}\right)‐({\text{ave}}_{f}\left((x_{g}‐\bar{x}){\;}^{2}\right)){\;}^{2}+4b^{2}\sigma_{g}^{2}}{4\tau^{4}}\\ & =\frac{3\sigma_{g}^{4}‐\sigma_{g}^{4}+4b^{2}\sigma_{g}^{2}}{4\tau^{4}}\\ & =\frac{\sigma_{g}^{2}\left(\sigma_{g}^{2}+2b^{2}\right)}{2\tau^{4}}, \end{array}$$

where the covariance equals zero because all central odd moments of the Gaussian distribution equal zero, and later making use of the fact that the uncorrected kurtosis of a normal distribution is three times the variance squared (Abramowitz & Stegun, 1972; see 26.1.18 and 26.1.26).

The partition of the variance in fitness into additive and nonadditive components is easily found, by calculating the slope across values of $x_{g}$ of mean fitness on trait from $cov_{f}\left(m_{g},x_{g}\right)=b\sigma_{g}^{2}/\tau^{2}$, and then the additive component as the squared slope times trait variance, to be

(14) $${\text{var}}_{f}\;m_{g}=\frac{\sigma_{g}^{2}\left(\sigma_{g}^{2}+2b^{2}\right)}{2\tau^{4}}=\frac{b^{2}\sigma_{g}^{2}}{\tau^{4}}+\frac{\sigma_{g}^{4}}{2\tau^{4}}.$$

The rate of change of mean fitness is established by differentiating Equation (11), and then substituting using Equations (12), (4) and (5) for terms in n, $\sigma_{g}^{2}$, and b, respectively, and then employing Equation (13), to find

(15) $$\frac{d{\text{ave}}_{f}m_{g}}{\mathrm{dt}}={\text{var}}_{f}m_{g}‐\frac{\lambda b}{\tau^{2}}‐\frac{{\text{ave}}_{f}m_{g}}{C}‐\frac{\mu^{2}}{2\tau^{2}}.$$

The final form in the text relies on the equality of the means of m and $m_{g}$, which was established in Equation (11), and on the partition just established into additive and nonadditive components of the genetic variance in fitness.
